# Policy Insights from High-Income Countries to Guide Safe, Nutritious, and Sustainable Alternative Proteins for Low- and Middle-Income Countries

**DOI:** 10.1016/j.cdnut.2023.101995

**Published:** 2023-09-03

**Authors:** Vivica Kraak, Mansha Kapur, Veena Thamilselvan, Anna Lartey

**Affiliations:** 1 Department of Human Nutrition, Foods, and Exercise, Virginia Polytechnic Institute and State University (Virginia Tech), Blacksburg, VA, United States; 2 The Johns Hopkins University, Baltimore, MD, United States; 3 Johns Hopkins Bloomberg School of Public Health, The Johns Hopkins University, Baltimore, MD, United States; 4 University of Ghana, Accra, Ghana

**Keywords:** policy, protein transition, alternative proteins, plant-based proteins, animal-source proteins, sustainable healthy diets

## Abstract

The United Nations has encouraged governments to promote sustainable healthy diets to address undernutrition, obesity, and climate change. This perspective paper examines policy insights from selected high-income countries in Asia, Europe, and North America to understand how traditional and novel alternative proteins (AP) may support sustainable healthy diets in low- and middle-income countries (LMIC) where populations experience malnutrition in all forms. AP products must be affordable, locally sustainable, and culturally acceptable to improve diet quality and health. Food-based dietary guidelines are a policy tool to guide AP product formulation, manufacturing, processing, labeling, and marketing to ensure that these products complement traditional plant- and animal-source proteins in sustainable healthy diets. This paper suggests that a new food categorization taxonomy is needed to guide AP product recommendations. Decision-makers must harmonize multisectoral policies to ensure LMIC populations have access to sustainable healthy diets to achieve a protein transition and food systems transformation by 2050.

## Introduction

The Global Syndemic encompasses 3 interacting pandemics associated with obesity, undernutrition, and climate change, which threatens human and planetary health [1]. Unsustainable food systems are a major driver of the Global Syndemic [1]. Synergistic multisectoral policies and actions implemented by diverse public- and private-sector actors are required to ensure future health for people, animals, the environment, and planet [1].

In 2022, the United Nations (UN) Secretary General and sixth International Panel on Climate Change (IPCC) urged governments to prioritize sustainable healthy diets to feed nearly 10 billion people by 2050, while mitigating the effects of climate change [2,3]. The IPCC report adopted the UN definition for sustainable healthy diets that “promotes all dimensions of individuals’ health and wellbeing; has low environmental impacts; and is accessible, affordable, safe, equitable and culturally acceptable” [3].

The FAO and WHO described 16 principles in 2019 to guide government actions for the health, environmental, and sociocultural domains to achieve sustainable healthy diets [4]. Promoting these principles will require coordinated, multisectoral policies tailored to the political economy, sociocultural contexts, and local and national food systems [4].

Businesses and civil society organizations have called for a global protein transition to rebalance protein consumption between conventional animal-source proteins and alternative proteins (AP) [5]. The protein transition aims to address concerns about the current industrialized agri-food systems linked to increased human diet and health risks, environmental degradation, animal welfare, workers’ rights and mistreatment, and the lack of affordable healthy diets for populations [6]. The AP paradigm shift aspires to decrease colorectal cancer and cardiovascular disease risks linked to diets with excessive red and processed meats (RPM) and reduce antibiotic resistance and zoonotic diseases spread from animals to humans [6,7]. The AP transition also aspires to reduce the environmental harms of large-scale, industrialized beef production associated with increased greenhouse gas emissions (GHGE), biodiversity loss, water pollution, and deforestation and to end the abusive treatment of farm workers and animals in confined feeding operations [[5], [6], [7]].

AP products and ingredients are derived from plant sources, such as edible insects (i.e., ants, caterpillars, crickets, grasshoppers), microorganisms (i.e., bacteria, fungi and yeast), algae, and cell-based agriculture, also called cultured or cultivated meat, poultry, fish, and seafood analogs [[7], [8], [9]]. Novel AP products tend to be higher in calories and sodium and contain many additives compared to traditional and fermented plant-based proteins (i.e., tofu, tempeh, and seitan) made from legumes (i.e., soybeans, chickpeas, lentils, fava beans), wheat gluten, fungi (i.e., mushrooms and yeast), and algae that have been consumed by diverse populations for centuries [[7], [8], [9]]. Thousands of novel AP products and ingredients have entered marketplaces worldwide since 2020, and research is underway to develop products that will sustainably feed humans by 2050 [[7], [8], [9]].

This perspective paper examines policy insights from selected high-income countries (HIC) in Asia, Europe, and North America that may be adapted to address the nutritional and socioeconomic considerations for culturally diverse low- and middle-income countries (LMICs). (We use The World Bank’s 2021–2022 country classification level for 4 income groups (i.e., low, lower-middle, upper-middle, and high-income countries (LMHIC) based on the gross national income (GNI) per capita of USD that include: $1085 or less in 2021 [LIC]; a GNI per capita ranging from USD $1086 to $13,205 [upper and lower MIC]; and a GNI per capita of USD $13,205 or higher [HIC] [10]). First, we examine expert recommendations for sustainable healthy diets. Second, we explore global trends and regional differences in animal-source compared with plant-based protein consumption. Third, we examine how AP products are addressed in national food-based dietary guidelines (FBDG) to inform policies for novel AP product development, formulation, processing, labeling, marketing, and regulation to align with sustainable healthy diets for all populations. Finally, we discuss HIC policy insights that may be considered within the context of economic sustainability and the future acceptability and affordability of next-generation AP ingredients and products to meet the needs of culturally diverse LMIC populations.

## Recommendations for sustainable healthy diets

In the context of growing noncommunicable diseases (NCDs) and GHGE from food systems, experts have recommended that HIC populations substantially reduce their intake of animal-source proteins, consume more plant-source whole foods (i.e., beans, pulses, legumes, nuts, and seeds), and adopt nutrient-dense planetary health diets [1,11]. The rationale for reducing RPM is that large-scale industrial beef production has a substantial environmental and climate footprint compared with plant-rich whole foods diets, measured by carbon footprints or GHGE, and land and water use [1,11]. Agricultural production, including food loss and food waste, contributes to 30% of global GHGE (i.e., methane and carbon dioxide) and 70% of freshwater use [1,11].

Most LMIC populations have different realities from consumers in economically affluent countries and do not consume the amounts of animal proteins suggested by the planetary health diet. These countries may experience a distinct protein transition [12]. LMIC have experienced a rapid nutrition transition as food systems have modernized and expanded access to ultraprocessed foods (UPF) and sugary beverages [13,14]. Consequently, women and children may experience the triple burden of malnutrition, which is the coexistence of undernutrition including stunting and micronutrient deficiencies (i.e., iron and vitamin A) and overweight and obesity in the same individual, family, or community [13,14].

Moreover, animal agriculture is deeply embedded in the cultural traditions of certain rural populations and can support sustainable livelihoods of families, regenerate soils, preserve biodiversity, and contribute to dietary protein and micronutrient adequacy to reduce malnutrition in vulnerable populations [15]. Therefore, governments must ensure that future food system policies address these factors as they consider incorporating novel AP products into national dietary guidelines and policies.

UN agencies have encouraged governments to adopt climate-smart agricultural practices, update dietary guidelines to integrate health and environmental sustainability principles, and promote affordable, nutrient-dense diets to address the Global Syndemic [1,11]. Guiding the effective inclusion of nutrient-dense AP could further promote healthy diets for HIC and LMIC populations [15]. Evaluating the country and regional context (i.e., geography, production practices, processing methods) that influence dietary intake is essential to tailor novel AP products to the nutritional needs of populations [15].

Many countries will not meet the UN’s global nutrition targets by 2025, exacerbated by the 2019 coronavirus pandemic, extreme weather attributed to climate change, and civil conflict. These factors will increase rates of acute and chronic forms of malnutrition [1]. Comprehensive policies are needed that address novel AP products tailored to the political economy, sociocultural contexts, and local food systems of countries to advance the UN’s Sustainable Development Goals 2030 agenda [1,4,5,13,16].

Growing evidence for the link between UPF, sugary beverages, poor diet quality and NCDs has led to the recommendation to avoid these products, including ready-to-heat and eat packaged and prepared convenience meals available in LMHIC markets [17]. Novel AP products are marketed as healthier and environmentally sustainable replacements to traditional animal-source proteins, despite limited evidence to support this claim [7,8]. Governments must develop comprehensive policies that balance many sustainability concerns to ensure that AP products will promote sustainable healthy diets and planetary health.

## Consumption of animal-source and plant-based proteins across regions

Global production and consumption trends of traditional animal-source proteins are important to understand the policy dilemmas faced by governments to restrict or replace these products and the market demand for plant-based AP meat and dairy products. Future policy and market scenarios are complex and may support either unsustainable business-as-usual practices or shift to more sustainable livestock production and consumption patterns within food systems, with varying impacts across regions [11,18]. Moreover, governments must balance many sustainability concerns (i.e., health, environment, and animal welfare) with economic prosperity, including trade policies [19].

This section briefly describes consumption trends for traditional animal-source proteins (i.e., RPM, fluid cow’s milk, and dairy) compared to plant-source proteins (i.e., AP meats and nondairy plant-based milks).

### Traditional animal-source proteins

Consumer demand for animal-source proteins varies by geographic region. However, affluent HIC populations in Europe and the United States have the highest intake of animal-source foods (i.e., meat and dairy), and HIC and upper-MIC populations have the highest RPM intake compared to the Southeast Asian and African regions [[18], [19], [20]]. Even if fluid milk and dairy intake decreased among HIC populations under a more sustainable production scenario, consumption of these products is projected to grow in the African and Southeast Asian regions [18].

### Plant-based and cultivated proteins

AP product expansion varies across countries and regions. The Good Food Institute and Euromonitor International reported that global 20222 retail sales of plant-based meat, seafood, milk and dairy was USD $28 billion [21]. Consumer demand for AP products is growing in the Asian Pacific, Australasian, Western European, and North American regions compared to Latin America, Middle East, Africa, and Eastern Europe; however, AP consumption trends reflect a niche market, representing a small percentage of the trillion dollar market for traditional animal-source proteins [21]. The global cell-cultured or cultivated meat market could reach USD $425 billion by 2030 but will depend on government regulatory approval (e.g., cultivated seafood is only approved for Singapore’s market and under consideration in a few countries), widespread affordability, and consumer acceptance of these products [22].

## HIC policy insights to guide AP products for LMIC populations

Current HIC policies to develop and regulate AP proteins are evolving and vary. Although some HIC have established specific AP regulatory bodies, many countries have yet to adopt select approaches. Government oversight and regulation of AP products has not kept pace with business innovation. Although AP manufacturing is rapidly expanding, there is no universally accepted classification system. The NOVA food classification system describes 4 categories for food and beverage groups and products based on level of processing (i.e., minimally processed, culinary ingredients, processed, and ultraprocessed) [17]. Many novel AP products would be designated as UPF using the NOVA food classification system and discouraged [17].

The Codex Alimentarius Commission is jointly coordinated by the FAO and WHO to develop guidelines, standards and voluntary codes of practice to protect consumer health while promoting international food trade [23]. However, the Commission has not recommended that governments use the NOVA food categories to define food quality for AP products [23]. No consensus exists for safe and healthy food processing and how novel AP products will address malnutrition, promote a healthy human microbiome, and support sustainable healthy diets [24,25]. Comprehensive policies and further research are needed to address these challenges.

Decision-makers must harmonize multisectoral policies to ensure that LMIC populations have affordable access to nutrient-dense diets, which include novel AP products, to prevent malnutrition. Table 1 and the next section discuss HIC policies for AP product development, manufacturing, processing, labeling, and marketing that may be adapted by LMIC decision-makers for the food system transition.

**TABLE 1:** Policy insights from selected HICs to guide safe, nutritious, and sustainable AP products for LMIC populations

Policies	General recommendations	Examples
Polices that support sustainable healthy diet principles and recommendations described in national FBDGs for populations.	Governments should update FBDG recommendations to encourage plant-rich sustainable diets that address many sustainability domains including human health, ecological health, and social equity and are available, affordable, and meet the needs of local populations [1,4,23,25,26,28]. Governments should clarify plant-based sustainable dietary patterns (i.e., Mediterranean, planetary health, vegetarian, flexitarian) and appropriate plant-based ingredient and product substitutions that provide similar nutrient-density profiles to animal-source proteins that do not exacerbate climate change [1,4,[24], [25], [26],^28^].	• HICs with FBDGs provide specific RPM intake targets that align with global expert recommendations to reduce diet-related NCD risks for *human health* (i.e., Australia, France, Italy, Mexico, and the United Kingdom) [26]. • Brazil, Canada, Denmark, Germany, India, Italy, Netherlands, Sweden, and United Kingdom provide FBDGs that encourage minimally processed, plant-based sources and/or environmentally sustainable diets that support *human and ecological health* [26]. • Argentina, Australia, Canada, China, Indonesia, New Zealand, and South Africa mention plant-based meat and dairy alternatives in FBDGs [28]. • By 2023, FAO reported that about 100 countries across 6 regions had developed FBDGs. LMIC governments could release FBDGs that address how AP substitutions could align with the FAO and WHO 2019 report guidelines for sustainable healthy diets [4].
Fiscal policies that support AP ingredients and products that are biologically safe, healthy for populations, and environmentally and ecologically sustainable for agri-food system supply chains.	Governments, businesses, and research institutions should collaborate to ensure adequate funding, investment in research and development, and fiscal policies (i.e., subsidies and taxes) that support AP ingredients and products that human and ecological health, social equity, and economic prosperity, including international trade policies [4,8,25,26,28].	• By 2023, Singapore had secured government approval of the commercial sale of lab-grown cultivated seafood; and the USDA had approved cultivated chicken, which require less space and resources than traditional proteins. The governments of China, Japan, Singapore, and South Korea have invested in research to develop and commercialize AP products given that Asia is the largest region with consumer demand [34]. • Israel, Singapore, and the United Kingdom established a single point of contact to establish the safety of novel AP product submissions and to increase transparency and clarify timelines for companies seeking approval for the marketplace [34].
Policies to guide AP ingredient and product manufacturing, formulation, processing and labeling.	Government regulatory agencies should update existing food classification systems and taxonomies to categorize novel AP products and provide guidance to manufacturers [24]. Governments should establish targets and timelines that require businesses to use nutrient-profile models and food classification systems to guide AP ingredient and product formulation, manufacturing, processing, and labeling that encourage consumers to select AP products with healthy nutrient profiles and lower ecological impacts than conventional animal-source proteins [30,35].	• The WHO provides nutrient-profiling models for Member States to guide policies, and more than 45 countries have enacted legislation for voluntary or mandatory FOP labeling [35] that industry can use for novel AP products to reduce sodium and excess calories through added sugars and fats and increase fiber and essential shortfall micronutrients (i.e., iron, calcium, and vitamins A, B, and D) to nourish populations [4]. • France launched the eco-score FOP labeling that provides information to consumers about the sustainability of food choices based on the product’s life cycle analysis [36]. • The European Union and United States have developed sustainable food profiling models that incorporate economic, environmental, and nutritional profiles into FOP labels to guide consumers’ choices [36].
Policies that guide accurate and non-misleading nutrition, health and environmental sustainability claims, marketing practices, and procurement policies for public institutions.	Governments should work with businesses and civil society organizations and seek stakeholder input to develop and adopt a simple, intuitive, universally recognized, and internationally and regionally applicable FOP labeling system that shows the nutrient and sustainability profiles for AP ingredients and products [36]. Governments should establish mandatory guidelines to restrict the marketing of AP products that do not meet nutrient-profile and ecological targets for human and ecological health [4].	• In 2023, the European Union established the Green Claims Directive to guide voluntary positive environmental claims made by businesses for products to prevent “greenwashing,” which is misleading or deceptive marketing targeted to consumers [37]. • In 2021, the United States Department of Agriculture announced a pilot school meals program to serve plant-based burgers to children, and the AP products may use soy or other vegetable protein sources with a biological quality of 80% milk protein determined by a protein digestible score [24]. • AP companies are using the legal system to challenge and amend labeling laws for AP products as producers of animal-source protein products aim to protect traditional labeling laws for milk and meat products marketed to consumers [24,34]. • In 2023, the United States Food and Drug Administration in the released guidance for industry to encourage transparent and accurate product labeling and nutrition and health claims recommendations for plant-based AP nondairy milk products. • In 2023, the European organization, ProVeg International, encouraged public procurement of plant-based AP products and works with local government, schools, and private caterers to provide menu consultations, recipe development, and impact assessments [38].

*Abbreviations:* AP, alternative protein; FBDG, food-based dietary guideline; FOP, front-of-package; HIC, high-income country; LMIC, low- and middle-income country; NCD, noncommunicable disease; RPM, red and processed meat.

### National FBDG policies

National FBDGs have been developed by more than 100 countries across the FAO’s 6 regions for Africa, Asia and the Pacific, Europe, Latin America, the Caribbean, and North America. National FBDG recommendations vary widely between countries. Many lack recommended targets for the dietary intake of specific food to meet the needs of vulnerable populations [26].

A 2022 review of the national FBDG recommendations for RPM intake for the G20 countries found that only 5 countries aligned their RPM targets in national dietary guidelines with expert recommendations to reduce NCD risks [26], and 6 countries recommended minimally processed, plant-rich choices and/or environmentally sustainable dietary patterns [26]. (The G20 countries were classified using The World Bank’s 2021–2022 country classification for LMHIC. The G20 countries include: 10 HIC [i.e., Australia, Canada, France, Germany, Italy, Japan, Republic of Korea, Saudi Arabia, United Kingdom, and United States]; 7 upper MICs [i.e., Argentina, Brazil, China, Mexico, Russian Federation, South Africa, and Turkey]; and 2 lower MIC [i.e., India and Indonesia] [27]). Indonesia, India and Saudi Arabia have the least red meat available for consumption (<10 kg per person annually), Turkey and South Africa have 14 to 18 kg per person annually, and the remaining G20 countries exceed the recommended red meat target (26 kg per person annually) [26]. MHIC governments could encourage populations to select plant-rich, sustainable healthy diets to reduce NCD risks [26].

A 2021 analysis of 100 FBDGs across 95 countries found that 38 guidelines had a position on vegetarian diets, and 45% mentioned plant-based alternatives to meat and milk. However, data gaps remain about how plant-based diets may balance the health, ecological, sociocultural, and economic factors that influence consumer behaviors [28].

FBDG often recommend cow’s milk as a high biological value protein source; culturally familiar animal livestock foods from goats, sheep, camel, and buffalo; and/or plant-based, fortified nondairy products that approximate the nutritional profile, taste, and texture of animal-source milks [28]. Expanding access to nutrient-dense AP products, as part of the protein transition, is important for LMIC populations [28].

AP dairy beverages marketed in HICs are derived from nuts and seeds, grains, legumes, and mixed-blends [29,30]. A large proportion of available nondairy beverages intended to replace milk are not nutrient-dense with adequate protein and micronutrients to match animal-source milk [30]. The market offers products lower in sugar and saturated fat compared to traditional milk products [29,30]. However, these beverages are often fortified with calcium but vary in protein (i.e., 0–10 g/serving), but not all are fortified with other essential vitamins and minerals found in animal-source milk [29,30].

Plant-based milks may have lower ecological footprints compared to traditional dairy; however, evidence is lacking for the large-scale human and environmental impacts of these AP products. Governments must develop standards and policies for the nutritional content and regulation for businesses to accurately market plant-based products as healthy and environmentally and economically sustainable in countries [29,30].

AP products or ingredients may support sustainable, healthy diets. The sources and proportion in people’s diets will depend on the specific product’s nutrient profile, bioavailability, consumer acceptance, and environmental impacts. National FBDGs must address the nutrition transition and enable consumers to understand how traditional and novel AP products may support sustainable healthy diets [[24], [25], [26],^28^,^31^]. Governments should translate FBDGs into graphic and simple text to communicate the what, why, how, and quantity to promote sustainable, healthy diets for populations [26,28,31].

### AP product development, formulation and processing

Policies are needed to guide novel product development (i.e., precision fermentation), formulation, and processing to remove excess calories from added sugars and fats, and sodium. Governments must establish and enforce mandatory targets and timelines for industry to develop novel AP products that improve the nutrient-density of AP products to be high in protein, fiber, and micronutrients and ensure that future products are nutritious, safe, affordable, acceptable, and have lower ecological footprints [32,33].

The current generation of AP products may reduce food safety concerns associated with traditional animal-source proteins including zoonotic diseases, microbiological contamination, and antibiotic resistance. However, the food industry must develop healthy and affordable products at scale that provide taste, texture, nutrient-density, and bioavailability of animal-source proteins; accommodate culturally diverse preferences for consumers who want minimally processed whole plant foods; minimize potential novel AP allergens; ensure affordable products for food-insecure populations; and ensure that AP ingredients and products do not adversely impact human health [33].

### AP product manufacturing, nutrient profiling, labeling and marketing policies

Only a few countries have developed technical recommendations to regulate AP products and ingredients. The governments of Denmark, Singapore, The Netherlands, Israel, Finland and France doubled their financial investments to advance AP products and technologies research during 2022 [34]. The regulatory environment for labeling and marketing in HICs, such as the United States, reveals tensions between producers and marketers of traditional animal-source and novel AP products [34].

The Codex Alimentarius Commission should develop guidelines for novel AP foods and products including: cultivated meat, fish, seafood and dairy; hybrid and plant-based products; insects; algae and microorganisms. UN agencies, global and regional advisory bodies should work with governments and national advisory bodies to develop international nutrient reference values and food safety, ingredient, and labeling standards. The Commission should develop guidance for health and nutrition claims to market traditional animal-source and novel AP products and harmonize international trade across countries [23,34].

More than 45 countries have adopted mandatory or voluntary, interpretive front-of-package (FOP) labeling, nutrient-profiling, and/or food classification systems to guide industry formulation and inform consumers’ selection of healthy food and beverage products [35]. These labeling and nutrient-profiling systems must be applied to novel AP plant-based products to meet sustainable healthy diet targets.

The current food classification systems and taxonomies are not adequate to categorize novel AP products and must be updated to clarify standards to support sustainable healthy diets [24,25]. A new food categorization taxonomy is needed to explain how novel AP products may complement traditional AP products to support sustainable healthy diets in diverse food system types. Although not widely applied, sustainable food profile modeling systems are available that score foods based on environmental profiles (i.e., GHGE, carbon footprint, or water use) and combine nutrition and environmental sustainability indicators including sustainable packaging [36].

Governments should also develop simple and universally recognized FOP labeling systems that depict nutrient and eco-sustainability profiles, accurate health claims, and guidance for AP products used in public procurement programs [[35], [36], [37], [38]] (Table 1). The HIC policy experiences may encourage decision-makers to develop standards for the processing, labeling, marketing, and regulation of novel AP products for LMIC populations.

Discussions about AP products have focused on novel plant-based and cultivated meat replacements among researchers, producers, and consumers in HICs but must engage LMIC researchers and consumers to determine relevance and acceptability of AP products for these regions. Decision-makers have opportunities to implement triple-duty actions to address the Global Syndemic. Although dietary affordability has improved over time in many countries, current food systems are not achieving nutrition and health outcomes, environmental sustainability, and equity targets [39].

The Food Systems Dashboard may consider including country AP production and policies to assist policymakers to monitor and evaluate performance to meet sustainable healthy diet and food system goals [39]. Novel AP products may contribute toward sustainable healthy diets in LMIC, but future research and policies must provide clarity on the nutritional content, safety, and environmental impacts. Future policies will depend on market opportunities for cultural acceptability and affordable approaches to meet the needs of diverse populations and the environment.

In conclusion, governments should update national FBDGs to clarify how novel AP products may support sustainable healthy diets. Decision-makers should tailor food system policies to address malnutrition in all forms including obesity and diet-related NCDs. LMIC populations need access to healthy nutrient-dense AP products to improve diet quality. However, novel AP products should be affordable, locally and regionally sustainable, and culturally acceptable. UN agencies should provide leadership and technical guidance to governments and businesses to reach consensus on a novel food categorization taxonomy that explicitly addresses novel AP products. Decision-makers could harmonize multisectoral policies for AP products to ensure that LMIC populations have access to nutrient-dense diets for the protein transition and food systems transformation by 2050.

## Acknowledgments

We thank Klaus Kraemer, Kesso Gabrielle van Zutphen, Jimena Monroy, and Jacquelyn Bedsaul at the *Sight and Life* Foundation for helpful feedback on earlier drafts and for coordinating the manuscripts included in the special issue.

## Author contributions

The authors’ responsibilities were as follows – VK: conceptualized the paper, wrote the first draft of the manuscript, and led the submission process; MK, VT, AL: contributed to the literature review; MK, VT, AL: provided input into the analysis and edited subsequent drafts; and all authors: read and approved the final manuscript.

## Conflicts of interest

AL serves as a reviewer of proposals on the Nestlé Foundation Council, an independent nonprofit organization. VK and AL have no relationship with the Good Food Institute. MK and VT voluntarily participate in an Alternative Protein Project at Johns Hopkins University that collaborates with the Good Food Institute.

## Funding

VK received partial funding from the Department of Human Nutrition, Foods and Exercise at Virginia Tech for salary support to research and write this paper. The *Sight and Life* Foundation provided funding to support the open access charges for this special supplement.

## Data availability

The data described in the manuscript, either abstracts or published papers that are open access, are available at the DOI provided after each citation in the reference section at the end of the paper. Other data relevant to codebooks are not relevant to this paper.
